# A Benign Outpouching Masquerading as a Scar: A Case of an Incidentally Diagnosed Cardiac Diverticulum

**DOI:** 10.7759/cureus.104861

**Published:** 2026-03-08

**Authors:** Ramin Tabibiazar, Samuel Daneshvar

**Affiliations:** 1 Cardiology, University of California Los Angeles, Los Angeles, USA

**Keywords:** cardiac diverticulum, cardiac mri, case report, echocardiography, left ventricular diverticulum

## Abstract

Cardiac diverticulum is a rare congenital anomaly characterized by an outpouching of a wall segment containing endocardium, myocardium, and pericardium. In contrast to a myocardial scar, there is an absence of fibrotic tissue, and this segment of myocardium contracts synchronously. Despite its rarity, proper recognition is important due to potential complications such as arrhythmia, heart failure, diverticular rupture, cardioembolic disease, and sudden cardiac death. The decision to treat is based on size, symptoms at presentation, and prognosis.

Our case involves a 51-year-old woman who was initially diagnosed with cardiac diverticulum in the basal segment of the left-ventricular inferior wall based on a transthoracic echocardiogram. Serial echocardiograms over the subsequent five years showed that the cardiac diverticulum has remained stable. She continues to do well clinically and has remained asymptomatic. The patient has required no cardiac intervention during this time interval.

## Introduction

Cardiac diverticulum is a rare congenital cardiac malformation characterized by an outpouching of the cardiac wall. This wall segment contains endocardium, myocardium, and pericardium [1]. In contrast to myocardial scars, there is no fibrosis in cardiac diverticula, and this segment contracts synchronously. It was first described in 1816 and remains a rare clinical finding, with a reported prevalence of less than 0.02% in autopsy series [1-4]. Although often asymptomatic and discovered incidentally, cardiac diverticulum carries clinical significance due to its potential complications, including arrhythmia, systemic embolization, heart failure, rupture, and sudden death [1]. The decision to treat depends on its size, associated symptoms, and prognosis [5]. In the absence of cardiac symptoms, follow-up intervals and serial echocardiograms may be required to monitor its growth, as intervention may need to be considered.

## Case presentation

**Figure 1:**
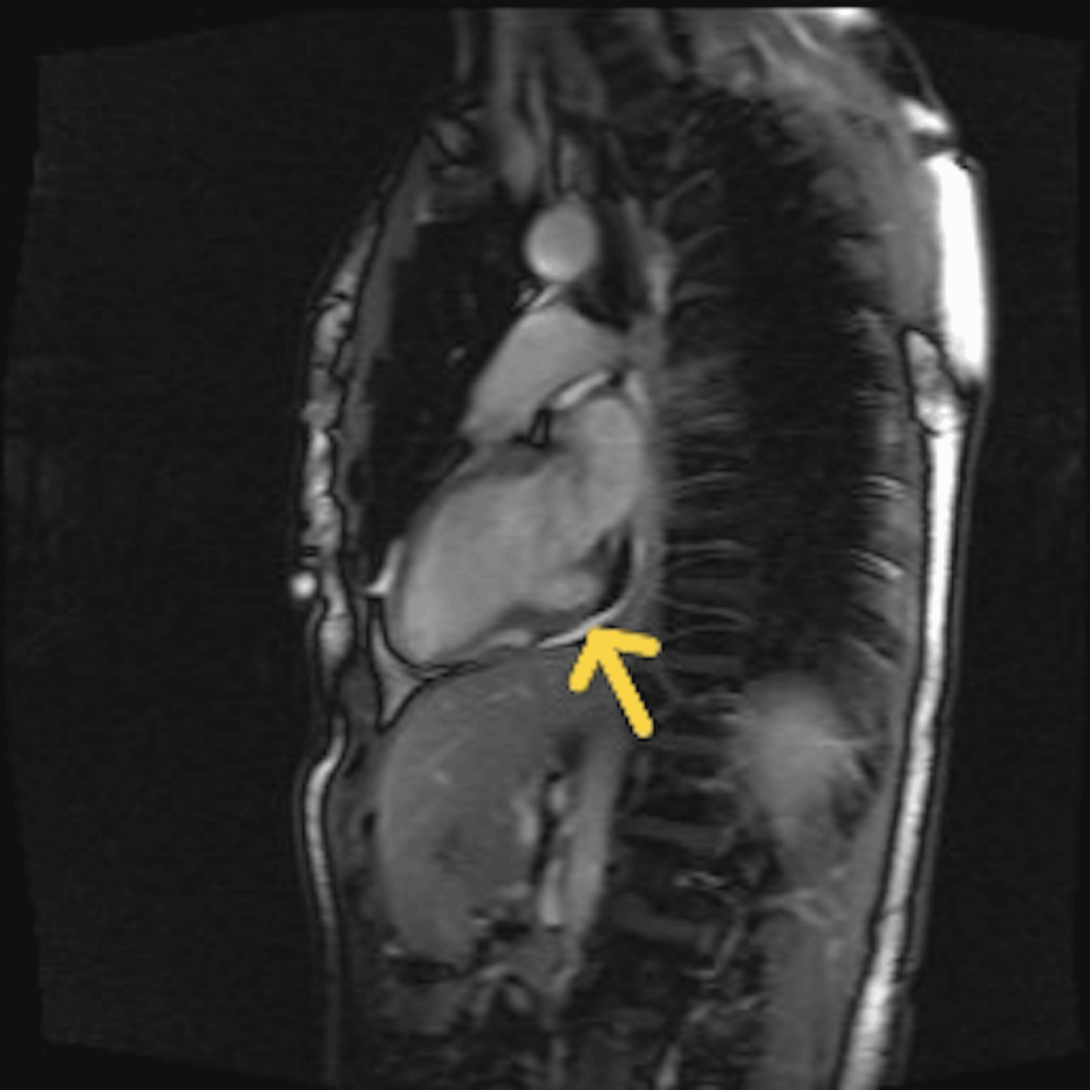
Cardiac magnetic resonance imaging (cMRI) showing cardiac diverticulum in the base of left-ventricular inferior wall during diastole (yellow arrow).

A 51-year-old woman underwent a transthoracic echocardiogram for further evaluation of atypical chest discomfort. The patient had no significant co-morbidities. Her chest discomfort occurred in the setting of worsening anxiety and depressed mood after the passing of her husband from myocardial infarction. The patient denied palpitations or syncopal episodes, and she did not have any prior history of cardiac events. She denied heart failure symptoms such as orthopnea, paroxysmal nocturnal dyspnea, or pitting edema. There was no history of tobacco, alcohol, or drug use. In addition, there was no history of trauma. Her vital signs were within normal limits, and her physical exam was unremarkable. Her baseline ECG showed a normal sinus rhythm with no abnormalities. Her echocardiogram showed an outpouching in the basal segment of the left ventricular inferior wall (Video 1). This outpouching segment measured 1.2 cm in size, and it showed preserved contractility (Video 1). Left-ventricular ejection fraction was preserved at 60-65%. She was diagnosed with cardiac diverticulum based on echocardiographic findings. Cardiac magnetic resonance imaging (cMRI) with gadolinium showed a focal outpouching without myocardial enhancement or scar, confirming the diagnosis of left ventricular diverticulum (Figure 1).

**Video 1:** Transthoracic echocardiogram (apical 2-chamber view) showing cardiac diverticulum in the basal inferior wall of left ventricle.

The patient's symptoms abated with supportive measures in the setting of recent bereavement. Her chest discomfort was attributed to anxiety related to bereavement and resolved with supportive care, confirming that the cardiac diverticulum was an incidental finding. She did not require any cardiac intervention or pharmacotherapy. The patient underwent serial echocardiogram examinations in the following five years. Her cardiac diverticulum has remained stable. She has remained asymptomatic from a cardiac standpoint, with no need for cardiac therapy.

## Discussion

Cardiac diverticulum is a rare condition. It was initially described in 1816 in Germany, and it was later reported in the United Kingdom in 1838 [1-3]. In 1912, the first case of surgical resection was reported [1]. Based on an autopsy series in children, the incidence of diverticulum is 0.02% [4]. Despite being a rare finding, the correct diagnosis remains important because of its potential harmful or even fatal complications [1].

Cardiac diverticulum involves the outpouching of a segment in the cardiac wall that histologically contains all three layers of wall. This outpouching segment contains endocardium, myocardium, as well as pericardium, and it contracts synchronously [1,5]. Cardiac diverticulum may occur as an isolated entity, or it may occur with other associated cardiac abnormalities or syndromes, such as Cantrell’s pentalogy, which is a condition with five defects: supraumbilical abdominal wall defect, lower sternal cleft, defect of the diaphragm in the central tendon, pericardium defect, and various intracardiac anomalies.

Cardiac diverticulum may affect apical or non-apical regions of the heart [5]. When it affects the apex, the diverticulum is usually a finger-like, contractile projection [5]. Apical diverticulum may be associated with midline thoracoabdominal defects, congenital heart disease, and dextrocardia or mesocardiac [5,6]. In general, apical diverticulum measures less than 3 cm in length and 1.25 cm in width [5,7]. It most commonly occurs in the left ventricle, but it can also occur in the right ventricle or both ventricles [5,8,9]. Similarly, non-apical diverticulum occurs more commonly in the left ventricle [5]. Non-apical diverticulum may also occur in the right ventricle, both ventricles, and/or atria [5]. Non-apical diverticulum is usually not associated with any other cardiac or congenital abnormalities [5,6]. While it may occur in various shapes and sizes, it usually ranges from 0.5 to 9.0 cm [5]. Non-apical diverticulum may have a narrow or wide neck that connects the outpouching to the cardiac chamber [5]. Table 1 summarizes the key features of apical and non-apical cardiac diverticula. 

**Table 1:** Comparison of apical and non-apical cardiac diverticula.

	Apical cardiac diverticulum	Non-apical cardiac diverticulum
Location	Most commonly occurs in the left ventricle, but can also occur in the right ventricle or both ventricles [5,8,9]	More commonly occurs in the left ventricle, but also occurs in the right ventricle, both ventricles, and/or atria [5]
Appearance	Usually a finger-like, contractile projection [5]	Various shapes [5]
Size	In general, measures less than 3 cm in length and 1.25 cm in width [5,7]	Varies in size. Usually ranges from 0.5 to 9.0 cm in size. It may have narrow or wide neck which connects the outpouching to the cardiac chamber [5]
Other associated defects	Midline thoracoabdominal defects, congenital heart disease, and dextrocardia or mesocardia [5,6]	None [5,6]

Left-ventricular diverticulum is considered to be congenital if there are no prior conditions that have injured the myocardium. The etiology of congenital apical diverticulum is thought to be due to failure in the fusion of the cardiac loop to the yolk sac [5,10]. Non-apical diverticula are caused by abnormal embryogenesis with a focal defect in the ventricular wall [11]. A congenital ventricular diverticulum can occur in a weak region of the ventricle wall within the first two or three weeks of embryonic life. Potential etiologies of the left ventricular diverticulum may include maldevelopment of the myocardium, intraventricular sinusoids, or viral infection that may result in a localized wall weakness with gradual contusion. 

Patients with congenital diverticulum are often not diagnosed at birth. Patients are frequently asymptomatic, and they are typically in their 30 to 60 years of age at the time of diagnosis [1,5]. In many instances, the condition is detected incidentally while undergoing diagnostic procedures [5,12]. Based on an angiographic study that examined ventriculograms, the prevalence of left ventricular diverticula in the adult population was 0.42%, with predominance in women [1]. Since the prevalence of cardiac diverticula was higher in the adult population than previously reported in children, whether all cardiac diverticula are truly congenital in origin has been questioned. It has been suggested that diverticulum of the cardiac free wall may develop later in adulthood due to increased intra-chamber pressure in combination with a congenital predilection [5]. Conditions that may lead to ventricular overload or high stress may contribute to the development of diverticula. These conditions include hypertension, heart valve disease, hyperthyroidism, obstructive hypertrophic cardiomyopathy, ventricular septal defect, atrial septal defect, or other congenital heart disease. In nearly 40% of patients with cardiac diverticulum, these associated conditions are present [5].

The diagnosis of cardiac diverticulum requires exclusion of underlying coronary artery disease, local or systemic inflammation, traumatic causes, and cardiomyopathies [1]. Cardiac diverticulum of the left ventricle needs to be distinguished from left ventricular aneurysm. Both conditions have distinct presentations, morphologies, and prognoses. Differentiating the two conditions is important given the therapeutic implications. In contrast to cardiac diverticulum, left ventricular aneurysm is a ventricular protuberance with akinetic or dyskinetic wall motion. Histologically, left ventricular aneurysms contain predominantly fibrous tissue, with no significant viable myocardium. 

Most patients with left ventricular diverticulum remain asymptomatic. In nearly 40% of the cases, patients may sustain systemic embolization, valvular regurgitation, infective endocarditis, heart failure, diverticular rupture, arrhythmia such as ventricular tachycardia, or sudden cardiac death [1,9]. In symptomatic patients, surgical resection may be required. The decision to treat is based on size, symptoms at presentation, and prognosis [5]. Spontaneous regression has been rarely reported [6]. Surgical resection may be performed if there is any need for cardiac surgery given associated congenital or acquired conditions. In the absence of associated cardiac abnormalities, the follow-up intervals are determined by symptoms. When the diverticulum increases in size, surgical intervention may need to be considered. Percutaneous closure using a patent ductus arteriosus (PDA) device has been reported in treating non-apical diverticula [13]. Anticoagulation therapy should be considered if there is systemic embolization [1]. When the patient has ventricular tachycardia, radiofrequency ablation or implantation of a defibrillator may need to be considered [1].

Our patient has been followed in the Cardiology clinic. She has remained stable, without active cardiopulmonary symptoms or cardiac events. She has not required any pharmacotherapy or cardiac interventions. The patient underwent two additional echocardiographic studies in the following five years, which demonstrated stability of her cardiac diverticulum. In addition, she had an ambulatory cardiac monitoring, which did not illustrate any sustained arrhythmia.

## Conclusions

Cardiac diverticulum is a rare but important clinical entity that must be distinguished from aneurysm due to differences in pathology and prognosis. Histologically, cardiac diverticulum involves an outpouching of a wall segment in the heart that contains endocardium, myocardium, and pericardium. There is an absence of scar tissue, and this segment contracts synchronously. While many patients remain asymptomatic and can be safely managed with surveillance, careful follow-up is essential to monitor for potential complications such as arrhythmia, systemic embolization, or diverticular rupture. This case highlights the value of multimodality imaging in diagnosis and demonstrates that stable, asymptomatic patients may not require intervention but benefit from long-term clinical monitoring. 
